# Pneumococcal Carriage in Children under Five Years in Uganda-Will Present Pneumococcal Conjugate Vaccines Be Appropriate?

**DOI:** 10.1371/journal.pone.0166018

**Published:** 2016-11-09

**Authors:** Ann Lindstrand, Joan Kalyango, Tobias Alfvén, Jessica Darenberg, Daniel Kadobera, Freddie Bwanga, Stefan Peterson, Birgitta Henriques-Normark, Karin Källander

**Affiliations:** 1 Department of Public Health Sciences, Karolinska Institutet, Stockholm, Sweden; 2 Public Health Agency of Sweden, Stockholm, Sweden; 3 Makerere University School of Public Health, Kampala, Uganda; 4 Sachs’ Children and Youth Hospital, Stockholm, Sweden; 5 Makerere University Faculty of Medicine, Kampala, Uganda; 6 Department of Microbiology, Tumor and Cellbiology, Karolinska Institutet, Stockholm, Sweden; 7 Department of Clinical Microbiology, Karolinska University hospital, Stockholm, Sweden; 8 Malaria Consortium, London, United Kingdom; 9 Department of International Maternal and Child health, Women´s and children´s health, Uppsala University, Uppsala, Sweden; 10 Ministry of Health, Kampala, Uganda; 11 Health and Demographic Surveillance Site, Iganga/Mayuge, Uganda; Universidade de Lisboa Faculdade de Medicina, PORTUGAL

## Abstract

**Background:**

Pneumonia is the major cause of death in children globally, with more than 900,000 deaths annually in children under five years of age. *Streptococcus pneumoniae* causes most deaths, most often in the form of community acquired pneumonia. Pneumococcal conjugate vaccines (PCVs) are currently being implemented in many low-income countries. PCVs decrease vaccine-type pneumococcal carriage, a prerequisite for invasive pneumococcal disease, and thereby affects pneumococcal disease and transmission. In Uganda, PCV was launched in 2014, but baseline data is lacking for pneumococcal serotypes in carriage.

**Objectives:**

To study pneumococcal nasopharyngeal carriage and serotype distribution in children under 5 years of age prior to PCV introduction in Uganda

**Methods:**

Three cross-sectional pneumococcal carriage surveys were conducted in 2008, 2009 and 2011, comprising respectively 150, 587 and 1024 randomly selected children aged less than five years from the Iganga/Mayuge Health and Demographic Surveillance Site. The caretakers were interviewed about illness history of the child and 1723 nasopharyngeal specimens were collected. From these, 927 isolates of *S*. *pneumoniae* were serotyped.

**Results:**

Overall, the carriage rate of *S*. *pneumoniae* was 56% (957/1723). Pneumococcal carriage was associated with illness on the day of the interview (OR = 1.50, p = 0.04). The most common pneumococcal serotypes were in descending order 19F (16%), 23F (9%), 6A (8%), 29 (7%) and 6B (7%). One percent of the strains were non-typeable. The potential serotype coverage rate for PCV10 was 42% and 54% for PCV13.

**Conclusion:**

About half of circulating pneumococcal serotypes in carriage in the Ugandan under-five population studied was covered by available PCVs.

## Introduction

Since 1990, the global under-five mortality rate decreased by 53%, from 91 to 43 per 1,000 live births [1]. While reductions in pneumonia, diarrhea, and measles collectively were responsible for half of the 3.6 million fewer deaths recorded in 2013 versus 2000, pneumonia still causes more than 900,000 deaths per year in children under five [2,3]. Globally, the estimated proportion of all child deaths attributed to pneumonia is 16% of which a third are due to *Streptococcus pneumoniae* (pneumococci) [2,4,5]. Essential interventions for pneumonia control include appropriate antibiotics in case-management, exclusive breastfeeding for the first 6 months and high coverage of childhood vaccinations against measles, *Haemophilus influenzae* type b and pneumococci [6,7].

Pneumococcal nasopharyngeal carriage is a prerequisite for invasive pneumococcal disease (IPD) [8] and the nasopharynx, particularly of young children, is considered the reservoir and the main source of pneumococcal transmission. A range of 35–93% of healthy children aged under five years in low-income countries has been shown to be colonized with pneumococci in the nasopharynx [9,10]. Pneumococcal conjugate vaccine (PCV) reduces vaccine-type pneumococcal carriage and thereby the dynamics of serotype transmission and pneumococcal disease [11]. Therefore, colonization studies pre- and post-implementation of PCV vaccination are important to obtain information on vaccine effectiveness [12,13].

Large-scale implementation of PCVs is ongoing in several African countries. Currently available PCVs include 10 or 13 serotypes out of at least 97 serotypes described so far. The serotype distribution differs with geographic area [14]. The serotypes included in PCV7 were chosen because they were the major serotypes causing IPD in the United States, but as PCV10 and PCV13 included serotype 1 and 5, the serotype coverage increased to also better cover invasive pneumococcal strains on the African continent [15]. However it is important to determine the serotype distribution in a country before introducing PCV in the childhood vaccination program [16]. In a recent systematic review on IPD and associated mortality and morbidity globally, serotypes 19F, 6B, 23F, 14 and 19A were the most commonly found disease causing strains in children below five years of age, all covered by PCV10 or PCV13 [17]. However, data from the African continent were scarce; only 42 strains were included in the analysis, which is too few to make any conclusions on potential vaccine effectiveness.

It is still unclear how effective PCVs will be in different African populations. Importantly, studies from South Africa, Kenya and Gambia show promising effectiveness [15,18–21]. In a study on patients with IPD in Uganda between 2003–2007 the potential vaccine serotype coverage was 55% for PCV7, 58% for PCV10 and 79% for PCV13 [22]. In a recent review on IPD serotypes in six West African countries the PCV10 serotype coverage was overall 68%, varying between 51–80%, except in Burkina Faso where PCV10 coverage was 39% [23]. In East Africa an IPD surveillance network published a study in 2009, showing a 56% IPD serotype coverage rate for PCV7 in children 6–29 months old [22].

In the African studies included in a systematic review from 2014, between 36% and 56% of the carried pneumococcal serotypes in children less than 5 years old were of PCV7-types, between 37–56% for PCV10 and 50–64% for PCV13 [9]. However, serotype distribution in both IPD and carriage varies over time and updated knowledge on pneumococcal serotype distribution in carriage is needed in order to inform policy choices and to access baseline data prior to the introduction of PCV10 in Uganda.

The aim of this study was therefore to study pneumococcal carriage and serotype distribution at multiple time points in a rural child population prior to PCV introduction in Uganda. We set out to determine the prevalence of pneumococcal colonization, the serotype distribution in the nasopharynx of healthy Ugandan under-five year old children at three time points and calculate the potential serotype coverage rates for PCV10 and PCV13 in carriage.

## Material and Methods

### Study population and area

**Fig 1:**
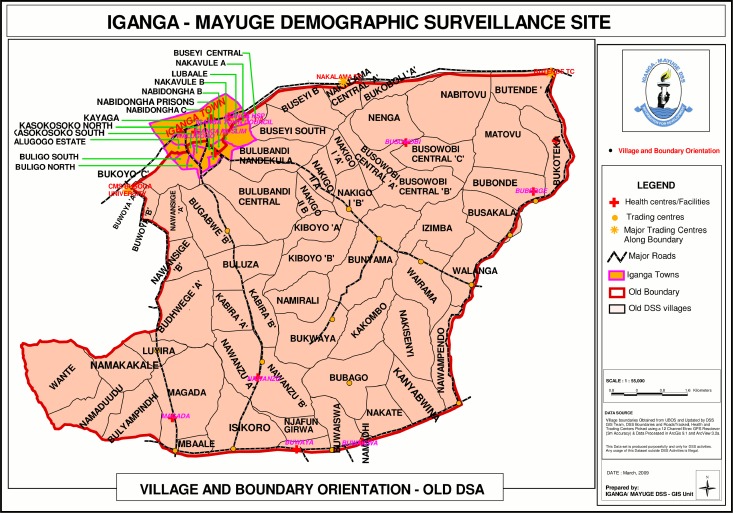
Map of the Iganga/Mayuge Health and Demographic surveillance site.

Three cross-sectional studies were carried out in 2008, 2009 and 2011 within the Iganga/Mayuge Health and Demographic Surveillance Site (HDSS), in East Uganda. Children under five years of age residing in the HDSS comprised the study population (Fig 1).

The HDSS comprises a population of 70,000 people, whereof approximately 11,000 are children under five years of age, living in a geographically defined area of in total 155 km^2^. The population is mainly rural with a few peri-urban areas and is served by 10 public health facilities (one hospital and 9 health centres), three non-governmental organization health facilities, and numerous drug shops and private clinics. In the HDSS births, deaths and information on migration and pregnancies are collected on a routine basis two times per year from each of the GPS coded households, following standard procedures recommended by the INDEPTH network [24].

Data from three independent surveys, nested within a cluster randomized trial (cRCT), were used for the analysis. The 2008 and 2011 surveys were powered to establish antibiotic resistance before and after a community intervention. The 2009 survey was powered to establish prevalence of pneumococcal carriage rates. In the cRCT, community health workers (CHWs) were trained and equipped to provide community case management of malaria (control) or malaria and pneumonia (intervention) for children aged 6–59 months [25]. Data on antibiotic resistance from these surveys is presented elsewhere [26].

### Sampling procedure and size

Sampling procedures differed between the three study years as the initial studies were not designed to combine data from the different surveys. For the surveys in 2008 and 2009 a random sampling of households with children aged under five based on probability proportion to village size was conducted using the HDSS database. For the follow-up survey in 2011 stratified random sampling was conducted for intervention and control areas under the cRCT. In the 2008 survey children between 2–59 months were included, whereas in 2009 and 2011 only children aged 6–59 months were recruited. In 2009 and 2011, all under-five aged children in a household were included and consequently the studies were considered cluster sampled. In 2008 one child was sampled per household using a lottery method. We sampled 152, 650 and 1400 children aged less than five years, in 2008, 2009 and 2011, respectively.

The sample size for the surveys in 2008 and 2009 were calculated using the formula for prevalence surveys, with finite population correction, n = Z_α_^2^(p)(1-p)/e^2^ [27], where Z_α_^2^ is the parameter related to the normal standard distribution, *p* the estimated prevalence and *e* the margin of error for the confidence interval. A Z-value of 1.96 (95% confidence) was used. For the 2008 survey assumptions were 10% margin of error and an estimated prevalence of pneumococci that were resistant to antibiotics equal to 50% (to get the most conservative sample size), giving a sample size of 96 children colonised with pneumococci to estimate the true resistance prevalence. Given that an estimated 66% of the under-five population is colonized by pneumococci [9] a total sample of 152 children was needed, allowing for a drop-out rate of 5%. For the survey in 2009, the sample size calculations were set to estimate the true prevalence of children carrying pneumococci and the assumptions used were p equal to 50%, 5% margin of error, and a cluster effect of 1.7 was added, yielding a total number of 650 children to be swabbed for bacteria. For the 2011 study, the sample size calculations of the cluster randomised trial were presented in the paper by Kalyango et al. [25] which generated a total sample of 1400 children under five after assuming a 25% non-response rate. Assumptions in 2011 used were 5% level of significance, 80% power, design effect of 1.9, 54% of children ill in previous 2 weeks, and a change in proportion of children receiving prompt and appropriate treatment of malaria from before the intervention (7.4–13.5%), giving a change rate of 13.5–24.3%, yielding a total number of 1094 children to be swabbed for bacteria.

### Data collection

A team consisting of one HDSS field assistant and one laboratory technician visited selected households and included sampled available children. Their caretakers were interviewed using structured questionnaires, and nasopharyngeal swabs were taken from the children. Field teams returned twice to the same household to include children not available at the first visit.

The pilot study in 2008 was performed in April during the rainy season, the study in 2009 in November by the end of the rainy season, and the 2011 study was done in January/February during the hot and dry season. The questionnaire was translated into the local language Lusoga and results were entered on a handheld personal digital assistant (PDA) in 2009 and on paper in 2008 and 2011. All symptoms were reported symptoms given by the care-giver. For each survey, the methods were taught and piloted during one day, respectively, with all field teams.

HDSS field assistants interviewed the main caretaker of the children aged under-five for any illness episode, care seeking and treatments used in the previous two weeks. Vaccination status was only asked in 2009. The household identifier was linked to the HDSS database for retrieval of household characteristics such as socio-economic and demographic factors.

### Specimen collection and transport

With caretaker consent nasopharyngeal swabs were taken by the technicians using a pre-packed sterile calcium alginate swabs on flexible aluminium shafts according to WHO guidelines [28,29]. Laboratory technicians were trained to use the correct technique to swab the back of the nasopharynx and the first author, who is experienced in the sampling technique, performed continuous training and supervision in the field. Swabs were placed in Amies transport medium in a tube and were transported to the Microbiology laboratory at Makerere University College of Health Sciences for primary culture, isolation and identification of *S*. *pneumoniae*. No specimen took longer than 12 hours to reach the laboratory.

### Laboratory analysis

The laboratory assays of identifying and isolating the bacterial isolates were performed as described in detail elsewhere [26]. In short, each swab was streaked on agar plates supplemented with 7% whole sheep blood and on chocolate agar. An optochin disk was placed in the first streak area and plates were incubated at 37° C in 5–10% CO_2_ for 24 to 48 hours. Pneumococci were identified by a zone of inhibition around the optochin disk and the presence of α-haemolytic, flat, irregularly shaped colonies with central depression with maturity. If two or more morphologically different pneumococcal colonies were identified, they were isolated separately. Pneumococcal isolates were stored at -70°C at the Microbiology laboratory at Makerere University Faculty of Medicine. The isolates were shipped on dry ice to the Public Health Agency of Sweden for pneumococcal serotyping by gel diffusion and/or Quellung reaction as previously described [30]. For the surveys 2008 and the first half of 2009, samples were shipped to Sweden in tubes with Amies transport medium, whereas the second half of 2009 and the 2011 specimen were sent frozen on dry ice.

### Variable definitions

Reported pneumonia symptoms were defined according to the WHO definition: cough and/or difficult breathing, and fast breathing (with or without fever). Upper respiratory tract infections were defined as cough or runny nose without fever, difficult breathing, or fast breathing. The definition for ‘child fully vaccinated’ was a child older than 20 weeks having had three doses of diphtheria, tetanus and pertussis (DTP) containing vaccine. Wealth quintiles were calculated using assets and household characteristics into a wealth index described elsewhere [31].

### Data management and analysis

Data from the questionnaires were downloaded from the PDA after the interviews, cleaned and coded by the data entry staff in the HDSS using FoxPro software in 2009. In 2008 and 2011 two members of HDSS staff independently manually entered the paper questionnaires data into FoxPro computer package. Excel was used to enter laboratory results. The questionnaires and the HDSS statistics were later transferred into STATA 12 and merged with the laboratory file into one file for analysis.

Descriptive statistics of frequencies of pneumococcal carriage, and pneumococcal serotypes were conducted and characteristics of the study participants in the three study years were compared using chi-squared (χ^2^) statistics. Bivariate analysis of individual characteristics (age, sex, vaccination status, history of illness and drug intake) and socio-economic factors (wealth quintiles) in relation to carriage of a resistant strain was also calculated using χ^2^ statistics or ANOVA. Logistic regression was used to evaluate risk factors for pneumococcal carriage. Variables with p-value<0.2 in the bivariate analysis were tested in the multivariable model. Odds rations (OR), p-values and 95% confidence intervals were calculated and p-values of less than 0.05 were considered significant. STATA version 12.0 was used for analysis.

## Ethical considerations

The study protocol was reviewed by the Institutional Review Board at Makerere University School of Public Health, and ethical approval was obtained from Uganda National Council for Science and Technology (Ref HS 72, HS 558, HS 559 and HS 898) and the Regional Ethics Committee in Stockholm (Ref 2010/1074-31/3 and 2011/1676-31/4). A material transfer agreement of specimen was obtained with the permission from the Uganda National Council of Science and Technology (Ref HS 72). Verbal consent was obtained from district and village leaders. Before data collection took place, an information form was read to the primary caretaker and data collection only started after the caretaker had given written or, if illiterate, a thumb-print, consent.

## Results

### Characteristics of the study population

In total 1761 children less than 5 years old were included, 150 in 2008, 587 in 2009 and 1024 in 2011. Overall 80% of the expected sample was achieved. The main reason for drop-out was absence at the time of the survey visits. Data on pneumococcal carriage was missing in 20 children in 2009 and in 18 children in 2011 due to refusal to sampling, failure to take a sample at the end of the interview or sample lost in the laboratory handling. A complete data set with serotype of carried pneumococcal isolate was available for 1723 children and was used in the final analysis.

The mean age was significantly lower in the year 2008 (25 months of age as compared to 33 and 36 months for year 2009 and 2011, respectively) since sampling included children from 2 months of age in this year (p = 0.02). The gender distribution between the sampling surveys was not significantly different (Table 1). Wealth quintiles were only available in 2009 and 2011, and the proportion in the poorest wealth quintile was higher in 2011 than in 2009, but the majority of households in both surveys were in the poorer to poor quintiles. Eighty-four percent of the children in 2009 were considered being on track with their vaccine schedule, i.e. being older than 20 weeks and having received three doses of DTP.

**Table 1:** Sociodemographic characteristics, illness and treatment in the last two weeks of children <5 year in the Health and Demographic Surveillance Site in Iganga/Mayuge district in Uganda in 2008, 2009 and 2011, n = 1761

Characteristics (number of children with missing data)	April 2008	Nov 2009	Jan/Feb 2011	P value
**Number of children**	150	587	1024	NA
**Age mean months (SD)** (0)	25	33	36	0.02
**Sex girls no. (%)** (0)	79 (53)	306 (52)	502 (49)	0.4
**Wealth quintile no. (%)** (201)				0.01
Poorest	*	67 (13)	159 (18)	-
Poorer	*	121 (23)	194 (22)	-
Poor	*	157 (29)	222 (25)	-
Less poor	*	105 (20)	195 (22)	-
Least poor	*	84 (16)	106 (12)	-
**On schedule for vaccinations no. (%)** (356)	*	193 (84)	*	NA
**Ill in last 2 weeks no. (%)** (1)	142 (95)	498 (85)	751 (73)	<0.001
**Pneumonia symptoms no. (%)** (78)	*	19 (3.2)	136 (13)	<0.001
**Other symptoms no. (%)** Fever (86)	131 (92)	431 (85)	707 (69)	<0.001
Runny nose (86)	87 (61)	386 (76)	708 (69)	0.001
Cough (86)	63 (44)	353 (69)	606 (59)	<0.001
Diarrhea (86)	24 (17)	65 (13)	236 (23)	<0.001
Vomiting (86)	14 (10)	40 (7.9)	196 (19)	<0.001
Difficult breathing (79)	*	32 (6.3)	148 (14)	<0.001
Fast breathing (78)	*	19 (3.7)	136 (13)	<0.001
Convulsions (78)	*	10 (2.0)	29 (2.8)	0.3
Other** (86)	9 (6)	49 (7.9)	60 (5.9)	0.3
**Treatment no. (%): any anti-malarials**	57 (38)	180 (31)	437 (43)	<0.001
Coartem (96)	15 (26)	69 (38)	332 (76)	<0.001
Chloroquine (96)	38 (67)	86 (48)	34 (7.8)	<0.001
Quinine (96)	7 (12)	35 (19)	77 (18)	0.55
Fansidar (96)	1 (1.7)	10 (5.6)	14 (3.2)	0.46
**Treatment no. (%): any oral antibiotics**	34 (23)	223 (38)	270 (26)	<0.001
Cotrimoxazole (96)	29 (85)	206 (92)	180 (67)	<0.001
Amoxicillin (96)	1 (2.9)	24 (11)	99 (37)	<0.001
Chloramphenicol (96)	2 (5.9)	8 (3.6)	2 (0.7)	0.006
Ampicillin (10)	5 (15)	6 (2.7)	8 (3.0)	0.01

*Question not included in the questionnaire for that year

**Other is skin rash, measles, wound, ear infection, typhoid, asthma, bleeding, abdominal pain, general weakness.

Reported illness in the last two weeks decreased from 95% in 2008 to 85% in 2009 and to 73% in 2011. Also the pattern of disease changed with reported fever decreasing from 92% to 69% and pneumonia symptoms increasing significantly from 2009 to 2011, from 3% to 13%. Difficult and fast breathing were not reported in 2008, and reported pneumonia could not be evaluated. Even if reported prevalence of fever was high, few children (between 31% and 43%) were reported to have received any antimalarial treatment. Between 23% and 38% of the children were reported to have received antibiotics within two weeks before the survey.

### Carriage rate and serotype distribution of *S*. *pneumoniae*

**Fig 2:**
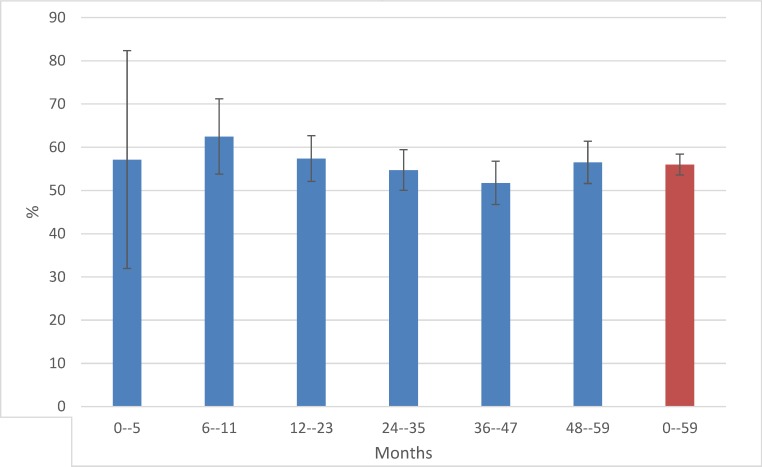
Pneumococcal carriage rate (%) by age group for children <5 years from Uganda, in 2008, 2009, 2011 combined (N = 957/1723)*. *Age group 0–5 months n = 8, 6–11 months n = 75, 12–23 months n = 194, 24–25 months n = 243, 36–47 months n = 201, 48–59 months n = 230, ages 0–59 months n = 957.

The overall pneumococcal carriage rate in children aged less than 5 years during the three survey years was 56% (957/1723; 38 missing observations). Carriage rates per year were 59% (88/150), 56% (315/567), and 55% (554/1006) in 2008, 2009 and 2011 respectively. The proportion of carriage varied from 52 to 63% across different age groups and was highest in the age group 6–11 months (Fig 2).

Of all children with pneumococcal carriage we were able to serotype at least one isolate in 89% (852/957) of the samples. One pneumococcal serotype was recovered from 777 (92%) of these children, while 75 (8%) of the children carried two serotypes. Children with more than 2 serotypes were excluded as laboratory handling problems (n = 6) and some strains did not grow after the transport to Sweden (n = 99). Altogether 927 pneumococcal isolates were serotyped from 852 children.

**Fig 3:**
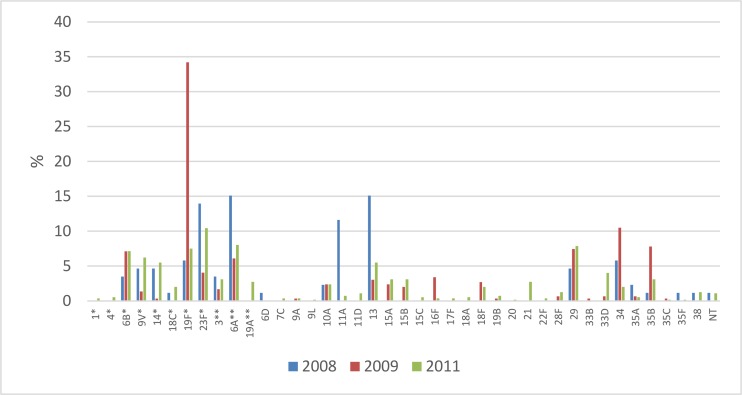
Proportion (%) of pneumococcal carriage serotypes from children <5 years in Uganda in 2008 (n = 86), 2009 (n = 295) and 2011 (n = 546).

The serotype distribution in 2008–2011 is shown in Fig 3. The most common serotypes for the three survey years combined were 19F (16%), 23F (9%), 6A (8%), 29 (7%) and 6B (7%). 19F was the most common serotype, mainly due to an increase in 2009. The most common non-vaccine types were 29 (7%), 13 (6%), 34 (5%) and 35B (4%). In total 40 different serotypes were identified.

**Fig 4:**
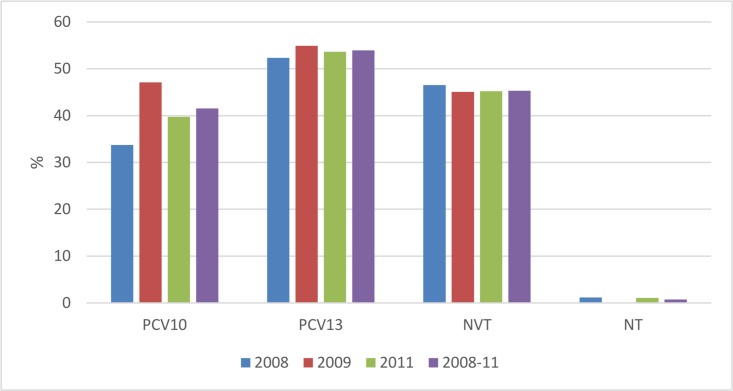
Proportion of pneumococcal carriage serotypes covered by pneumococcal conjugate vaccines from children <5 years in Uganda, 2008, 2009 and 2011 (n = 927)*,**, ***. *The PCV13 bars includes the PCV10 bar + the three extra serotypes (3, 6A and 19A). **NVT is non-vaccine types. ***NT is non-typable

The proportions of strains covered by PCVs in 2008–2011 were 42% for PCV10 and 54% for PCV13. Only 1% of the strains were non-typeable. 58% and 45% were non-PCV10 and non-PCV13 vaccine types (NVT), respectively (Fig 4).

### Risk factors for carriage

To analyze risk factors for pneumococcal carriage, bivariate and multivariable logistic regression were performed (Table 2). In the bivariate analysis we found that being ill in the last two weeks and being ill on the day of the sampling were significantly associated with an increased odds ratio for pneumococcal carriage (p = 0.02 and 0.04, respectively), while higher age was borderline significantly associated to a decreased odds ratio for pneumococcal carriage. The final model, adjusted for gender, age in months, ill in the last two weeks, and ill on the day of the interview showed that only ill on the day of the interview was a significant predictor of pneumococcal carriage (OR 1.50; 95% CI 1.03–2.19; p = 0.04).

**Table 2:** Factors associated with pneumococcal carriage in children <5 years in Uganda in 2009 (n = 587, missing = 20) and 2011 (n = 1024, missing = 18)*.

Variable	Category	Unadjusted			Adjusted **		
		OR	95% CI	P	OR	(95% CI)	P
**Age (n = 1573)**	months	0.99	0.99–1.00	0.06	0.99	0.99–1.00	0.46
**Sex of the child (n = 1573)**	Female	0.99	0.81–1.21	0.95	0.99	0.71–1.38	0.94
	Male	Ref.			Ref.		
**Wealth quintile (n = 1379)**	Poorest	0.88	0.60–1.72	0.54			
	Poorer	0.96	0.68–1.37	0.83			
	Poor	0.89	0.64–1.25	0.51			
	Less poor	0.93	0.66–1.33	0.71			
	Least poor	Ref.					
**On schedule for vaccinations (in 2009) (n = 231)**	No	0.97	0.50–1.88	0.92			
	Yes	Ref.					
**Ill in 2 w. before interview (n = 1573)**	No	Ref.			Ref.		
	Yes	1.28	1.01–1.63	**0.04**	0.90	0.54–1.51	0.69
**Ill on the day of interview (in 2009) (n = 566)**	No	Ref.			Ref.		
	Yes	1.48	1.05–2.08	**0.02**	1.50	1.03–2.19	**0.04**
**Upper respiratory infection (n = 1573)**	No	Ref.					
	Yes	1.28	0.86–1.93	0.22			
**Pneumonia symptoms (n = 1573)**	No	Ref.					
	Yes	1.06	0.81–1.39	0.66			
**Fever (n = 1498)**	No	Ref.					
	Yes	1.17	0.93–1.47	0.19			
**Any antibiotic in the last 2 weeks (n = 1573)**	No	1.04	0.84–1.30	0.68			
	Yes	Ref.					
**Any antimalarial in the last 2 weeks (n = 1573)**	No	1.0	0.82–1.23	0.96			
	Yes	Ref.					

* Unadjusted and adjusted odds ratios (OR), 95% confidence intervals (95% CI) and P-value (P)

**Multivariate logistic regression, adjusting for the other factors shown in the table column

For access to the full data set, please contact: Ann.Lindstrand@folkhalsomyndigheten.se

## Discussion

These repeated cross-sectional surveys show that vaccine serotypes represented about half of all serotypes carried in Ugandan children younger than five years of age, during three years preceding the implementation of a national pneumococcal conjugate vaccine program. The potential serotype coverage of PCV10 was 42% and 54% for PCV13 for colonizing pneumococci.

The overall pneumococcal carriage rate was 56% in our study. In a systematic review on pneumococcal carriage in Sub-Saharan Africa[10], carriage rates varied widely between 21–94%, but in the meta-analysis used, carriage rates (63%) were similar to those in our study.

As the burden of pneumococcal disease in Uganda is high [25,32] and management of pneumococcal disease is inadequate with regards to both access to and quality of case management [25,31,33], the implementation of the PCV program for children that was launched in 2013 is therefore important to prevent child deaths and morbidity due to pneumococcal disease. A previous study estimated that a PCV program in Uganda with 79% vaccination coverage and vaccine protection against 63% of invasive serotypes would be cost-effective, and would each year save more than 10,000 lives and prevent 94,000 cases of infections caused by *S*. *pneumoniae* in children less than 5 years old in Uganda [34]. Our study on colonizing strains show that the PCV10 vaccine used in Uganda has a lower serotype coverage in carriage compared to IPD serotype coverage (42%), as has been shown in other countries [35–37].

In Uganda, a study using data from 2001–2006 showed that a third of all children less than 5 years old with bacterial meningitis had positive culture for *S*. *pneumoniae*, with an incidence of pneumococcal meningitis between 3-42/100,000. Of the positive cultures, 40% were of serotype 6A or 6B, and 43% of the serotypes were included in the PCV7, 46% in PCV 10 and 70% in PCV13 [38]. Even though only 30 of the strains were serotyped, these results resemble our data on PCV coverage for colonizing pneumococci. In a larger study from four East African countries including Uganda, typing of 442 invasive pneumococcal strains showed a 56% coverage for PCV7, 70% for PCV10 and 79% for PCV13 in children less than 5 years of age sampled in 2003–2007 [22]. These and other studies show a higher coverage of PCV10 and PCV13 among pneumococcal isolates causing invasive disease than we show for carriage isolates. It may be due to a different serotype distribution in colonizing bacteria compared to those causing invasive disease (such as serotype 1 and 5 [39]). Colonizing pneumococcal strains may initiate protective immunity better and therefore be less prone to cause invasive disease [40].

Some study limitations are acknowledged. First, data collection during the three years was done at different times of the year with different seasonality. While our main outcome, pneumococcal carriage rate, remained relatively stable over the three years, the different seasons may explain the difference in reported disease, with more fever (potentially due to malaria) in the rainy season (92% and 85% vs. 69%) and more pneumonia (22% vs. 8%) in the dry season. Secondly, the 2011 study collected data two years after the introduction of a cRCT on community case management of pneumonia and malaria where the intervention could have changed the carriage rate due to more use of amoxicillin in the intervention villages. However, no significant difference was noticed in carriage rates among children in the control (53%) and intervention villages (55%) (p = 0.06) in 2011.

Thirdly, logistic problems, including delayed flights, occurred during the shipping of samples transported in room temperature from Uganda to Sweden, and therefore some isolates had to be sent twice before they could finally be serotyped. Despite this, we managed to serotype 89% of the samples. One could speculate that some more sensitive strains of certain serotypes died selectively in the transport, resulting in a biased serotype representation. However, the serotype distribution was similar across the three years except for a peak of 19F in 2009. Also, as a result of not having reached the anticipated sample size we may have lost power to detect statistically significant differences between variables. Additionally, reported illness and treatment in the last two weeks were collected from caregivers and results may suffer from recall bias. The differences in symptoms between surveys, for example a history of fever and pneumonia, were large, even though similar questionnaires with identical symptom questions were used in the surveys. Further research would be required to understand if this is related to seasonality, changing epidemiology, or simply inconsistency in how the questions were asked. Some known risk factors for pneumococcal carriage were not included in the questionnaire, such as HIV status, smoking in the family, number of siblings, and could therefore not be evaluated or compared to other studies [41,42].

Finally, we did not perform any molecular analysis other than serotyping. While the same serotype can be more or less invasive due to genetic differences in clones [43], the capsular serotype is still the major virulence factor [44,45].

The serotype coverage found in our study is similar to some other studies from the African continent. A study in Gambia showed a potential PCV13 coverage of 47% in children under 5 years before PCV13 implementation [46]. In Kenya, carriage by PCV10 serotypes, was lower than in our study, and was further reduced by two thirds after PCV10 introduction, from 34% to 13% [47]. In Mozambique serotype 1 and 5 were common in IPD but not in carriage, showing that some serotypes with high invasive disease potential colonize the nasopharyngeal space for a shorter time span [48]. In Senegal, serotype 6B, and 23F were common in both IPD and carriage, but serotype 1 was most prevalent in invasive disease [35]. However, PCV effectiveness studies, so far, in Africa are still promising. In South Africa, incidence of IPD in children less than 2 years decreased by 89% (95% CI 86–92) [18].

### Conclusion and implications for policy and practice

Half of circulating pneumococcal serotypes in carriage in the Ugandan under-five population was covered by available pneumococcal conjugate vaccines. These data give a measurement of the potential vaccine effectiveness against pneumococcal carriage, and baseline data for deciding which vaccine to be used in Uganda. More research is needed to evaluate the effectiveness of PCV on incidence of pneumococcal disease, including future vaccination coverage, while it is being implemented over the coming years in Uganda and other East African countries.
